# Small bowel obstruction outcomes according to compliance with the World Society of Emergency Surgery Bologna guidelines

**DOI:** 10.1093/bjs/znaf080

**Published:** 2025-04-18

**Authors:** Lewis J Kaplan, Isidro Martinez-Casas, Shahin Mohseni, Matteo Cimino, Hayato Kurihara, Matthew J Lee, Gary A Bass, SnapSBO Collaborators, Agron Dogjani, Kastriot Subashi, Klevis Doci, Joana Spaho, Ali Abdulla, Sara Ahmed, Yusuf AlAnsari, Mariam AlKooheji, Alaa Marzooq, Khaled Nazzal, Emir Ahmetašević, Zlatan Mehmedović, Maja Kovačević, Jasminka Mujkanović, Peter Svenningsen, Marie Peter Møller, Gitte Emilje Olsen, Abeer Aboalazayem, Muhammad Ashrad Awad, Mahmoud M A Elfiky, Moemen Farouk, Mostafa Gad, Basma Magdy, Peep Talving, Edgar Lipping, Edgar Lipping, Sten Saar, Artjom Bahhir, Maarja Talviste, Vincent Dubuisson, Luca Cigagna, Luigi Cayre, Spyridon Christodoulou, Panagiotis Kokoropoulos, Ioannis Margaris, Maria Papadoliopoulou, Theodoros A Sidiropoulos, Panteleimon Vassiliu, Evangelos Barkolias, Pavlos Georgalis, Theodosios Kantas, Vasiliki Nikolaou, Aristeidis Papadopoulos, Katerina Tata, Stergios Arapoglou, Ioannis Gkoutziotis, Aikaterini Mpratko, Elissavet Symeonidou, Stylianos Kykalos, Nikolaos Machairas, Adam Mylonakis, Panagiotis Sakarellos, Dimitrios Schizas, Michail Vailas, Iraklis Anastasiadis, Parmenion Patias, Koumarelas Konstantinos, Mourtarakos Saradis, Charles Lee, Chloe Spillane, Dylan Viani Walsh, Nadia Walsh, Thomas Noel Walsh, Gabriel Orsi, Andrew Keane, David Kearney, Emma de Sousa, Michael Sugrue, Anne Marie Doyle, Robert Fitzsimmons, Angus J Lloyd, Mohammad Saad Qasim, Mashood Ahmed, Taylor Jacoby, Michael E Kelly, Shafagh Khodadi, Paul McCormick, Éanna J Ryan, Mahmoud M Salama, Helen Heneghan, Cian Davis, Odhran K Ryan, Sean T Martin, Miklosh Bala, Michele Altomare, Stefano P B Cioffi, Andrea Spota, Giada Panagini, Laura Benuzzi, Stefania Cimbanassi, Noemi DiFuccia, Stefano Manfroni, Alan Biloslavo, Paola Germani, Nicolo de Manzini, Manuela Mastronardi, Anna Modica, Serena Scomersi, Gabriele Bellio, Luigi Cayre, Gaia Altieri, Pietro Fransvea, Gabriele Sganga, Silvia Tedesco, Francesca Bunino, Sabrina Caspani, Daniele DelFabbro, Simone Giudici, Giulia Mauri, Paolo Meneghesso, Enrico Ortolano, Antonella D'addiego, Francesca Di Vittorio, Gabriele Bormolini, Michele Carlucci, Giovanni Pesenti, Claudia Tintori, Mauro Zago, Martina Zambon, Simona Meneghini, Andrea Mingoli, Giulia Duranti, Gioia Brachini, Pierfrancesco Lapolla, Mehdi Hanafi, Clara Valdez Cruz, Andrea Alfredo Huerta de León, Jose García Regalado, Pasquale de Jesús Cristiano Nakhal, Diego Enrique Rodríguez González, Jose Ruiz, Salvador Lozada Jimenez, Oscar Carlos Delgado, Monserrat Reyes Zamorano, Anyely Fuertes Muñoz, Ademola Adetoyese Adeyeye, Ehis Afeikhena, Akinola Akinmade, Babatunde Mustapha, Jaroslav Presl, Patrick Rebnegger, Bjoern Rudisch, Gruenfelder Johanna, Rokitte Karin, Filipa M CorteReal, Jorge A Pereira, Joao L Pinheiro, Daniela M Pinto, Andreia J Santos, Andreia M Silva, Susana Henriques, Joao Melo, António Miguel Pereira, Antonio Miguel Pereira, Ana Margarida Cabral, Bruno Dias Couto, Barbara Nunes Gama, Catarina Santos Rodrigues, Mara Nunes, Bruno Ribeiro Silva, Daniela Tavares, Daniela Tavares, Toma Mihai, Oprea C Valentin, Srdjan S Putnik, Petar Andjic, Marija Djujic, Rastislav Filko, Vanja Kunkin, Andjela Milak, Aleksandar Ognjenovic, Nebojsa Mitrovic, Goran Aleksandric, Mihailo Bezmarević, Sasa Dragović, Milan Jovanović, Bosko Milev, Miroslav Mitrović, Srdjan Petković, Valentina Isakovic, Nikola Zoran Nikolic, Predrag Radic, Dragan Luka Vasic, Zlatibor M Loncar, Dusan D Micic, Vladimir R Resanovic, Pavle D Vladimir, Krstina S Doklestic Vasiljev, Ljiljana Velibor Milic, Vladica Velibor Cuk, Jovan Todor Juloski, Radisav Slavoljub Radulovic, Dragana Dragan Arbutina, Jacobo Trebol, Manuel Torres-Jurado, Andres J Valera-Montiel, Francisco E Blanco-Antona, Beatriz de Andrés-Asenjo, Maria Ruiz-Soriano, Tania Gómez-Sanz, Andrea Vázquez-Fernández, Juan Beltran de Heredia, Cristina Rey-Valcárcel, Monica Ballón-Bordon, Maria Pérez-Díaz, Maria Dolores Sanchez-Rodriguez, Jose David Gonzalez-Esteban, Celia Alegre Nevado, Ricardo Montenegro Romero, Andrea Campos-Serra, Raquel Gracia-Roman, Heura Llaquet-Bayo, Anna Muñoz-Campaña, Giulia Vitiello, Lorena Apodaca Murguiondo, Inigo Augusto Ponce, Amaia Garcia Dominguez, Aintzane Lizarazu Perez, Elena Sagarra Cebolla, Mónica García Aparicio, Paloma Garaulet González, Benito Miguel Josa Martínez, Miriam Fraile Vasallo, Mónica MengualBallester, Isabel Andrés Lucas Zamorano, Jose Martinez Moreno, Manuel Luis Buitrago Ruiz, Clara Piñera Morcillo, Alberto Díaz García, Hanna Hernández Oaknin, Maria Pellicer Barreda, Jennifer Amparo García Niebla, Antonio Pérez Álvarez, Diego Cordova, Laura Jiménez, Fernando Mendoza, Cristina Vera, Alberto Vilar Tabanera, María de los Ángeles Gil-Olarte Márquez, José Antonio López-Ruiz, Mª Estela Romero-Vargas, Julio Reguera-Rosal, Alberto García-García, Beatriz Marenco de la Cuadra, Eduardo Perea del Pozo, Virginia Duran Muñoz, Felipe Pareja Ciuró, Ainoa Benavides dos Santos, Ernest Bombuy, Anna G-Monferrer, Sandra López Gordo, José Guerra, Vanessa Sojo, Begona De Soto, Aaron Roman, Ana María González-Castillo, Elena Manzo, Estela Membrilla-Fernandez, Amalia Pelegrina-Manzano, Simone Cremona, Alexander Forero-Torres, Santiago Valderrabano, Francisco Reinoso Olmedo, Fuad Lopez Fernandez, Aitor Landaluce-Olavarria, Jon Barrutia- Leonardo, Alba Garcia-Trancho, Melania Gonzalez-De Miguel, Izaskun Markinez-Gordobil, Maryam Makki, Dana Altamimi, Sadhika Vinod, Olga Rutka, John V Taylor, M Denton, S Gourgiotis, R Ravi, A J Ribbits, Jared Wohlgemut, Shehryar Rangana Khan, Christopher Leiberman, Sabreen P Elbakri, Charlie A Edgar, Conor Magee, Oluwaseun Oyekan, Mehwish Ansar, Jeremy Wilson, Rahel Rashid, Deborah Atwell, Joshua Cassedy, Brianna Gabriel, William Hoff, Shyam Murali, Anna E Garcia Whitlock, Carolyn Susman, Sarah Barnett, Emily Ertmann, Camden DeSanctis, Pavel Karasek, Nathan Klingensmith, Dale F Butler, Brandon Bruns, Ankeeta Mehta, Vanessa Nomellini, Keyus Patel, Anthony Tannous

**Affiliations:** Division of Traumatology, Surgical Critical Care, and Emergency Surgery, Perelman School of Medicine, University of Pennsylvania, Philadelphia, Pennsylvania, USA; Unidad de Cirugía de Urgencias y Trauma, Hospital Universitario Virgen del Rocio, Sevilla, Spain; School of Medical Sciences, Orebro University, Orebro, Sweden; Department of Emergency Surgery, Fondazione IRCCS Ca’ Granda Ospedale Maggiore Policlinico, Milan, Italy; Department of Emergency Surgery, Fondazione IRCCS Ca’ Granda Ospedale Maggiore Policlinico, Milan, Italy; Institute for Applied Health Research, University of Birmingham, Birmingham, UK; Division of Traumatology, Surgical Critical Care, and Emergency Surgery, Perelman School of Medicine, University of Pennsylvania, Philadelphia, Pennsylvania, USA

## Abstract

**Background:**

Small bowel obstruction (SBO) is a common surgical emergency associated with substantial morbidity, hospital length of stay (LOS), and healthcare cost. The World Society of Emergency Surgery (WSES) Bologna guidelines provide evidence-informed recommendations for managing adhesive SBO, promoting timely surgical intervention (or non-operative management (NOM) when ischaemia, strangulation, or peritonitis are absent). However, guideline adoption and its impact on outcomes remain under studied. Compliance with the Bologna guidelines was evaluated to determine the impact of compliance on outcomes.

**Methods:**

SnapSBO, a prospective, multicentre, time-bound, observational cohort study, captured data on patients with adhesive SBO across diverse healthcare settings and patient populations. Patient care was categorized into: successful NOM, surgery after an unsuccessful appropriate trial of NOM (NOM-T), and direct to surgery (DTS). Compliance with diagnostic, therapeutic, and postoperative Bologna guideline recommendations was assessed as either complete or partial. Primary outcomes included adherence to the Bologna guidelines, LOS, complications, and the incidence of the composite metric ‘optimal outcomes’ (LOS ≤5 days, discharge without complications, and no readmission within 30 days).

**Results:**

Among 982 patients with adhesive SBO, successful NOM occurred in 561 (57.1%), 224 (22.8%) underwent NOM-T, and 197 (20.1%) proceeded DTS. The mean(s.d.) LOS was 5.3(9.0), 12.9(11.4), and 7.7(8.0) days respectively (*P* < 0.001). Optimal outcomes were achieved in 61.0%, 16.1%, and 37.6% respectively (*P* < 0.001) and full guideline compliance was observed in 17.2%, 10.1%, and 0.4% respectively.

**Conclusion:**

Patients with adhesive SBO whose care was aligned with the Bologna guidelines had a shorter LOS and a greater incidence of optimal outcomes. Addressing evidence-to-practice gaps through implementation strategies that consider contextual factors will enhance guideline adoption and patient outcomes.

## Teaser

The multinational prospective study SnapSBO evaluates the real-world impact of adherence to the WSES Bologna guidelines on adhesive small bowel obstruction outcomes. Across 70 centres in 20 countries, higher compliance correlates with reduced length of stay and improved patient outcomes, yet full adherence remains infrequent, particularly in operative cases. These findings highlight persistent evidence-to-practice gaps and underscore the need for targeted implementation strategies to enhance guideline adoption and optimize adhesive small bowel obstruction management.

## Introduction

Small bowel obstruction (SBO) is a common cause of surgical admission and is associated with significant morbidity, healthcare cost, and resource utilization^1–3^. Adhesions are the most common aetiology in high-income settings, whereas malignancy and hernia are more globally represented^4,5^. Despite diagnostic, therapeutic, and technical advances, patients continue to face delayed symptom resolution, high complication rates, and frequent recurrence of obstruction^6,7^.

Evidence-informed expert consensus guidelines aim to reduce practice variability, enhance treatment effectiveness, and improve clinical and patient-reported outcomes. The World Society of Emergency Surgery (WSES) Bologna guidelines offer recommendations encompassing diagnosis, non-operative management (NOM), surgery, and postoperative care for patients with adhesive SBO^3^. See *Table 1*.

**Table 1:** Summary of key recommendations from the WSES Bologna guidelines (2017 update) for the management of adhesive SBO

WSES Bologna guideline recommendation statement	Supported treatment pathway			Level of evidence	Supporting evidence
	DTS	Successful NOM	NOM-T		
Presence of clinical signs of peritonitis or CT findings (pneumatosis, closed-loop obstruction, perforation, or bowel ischaemia).	✓	✗	✗	IIC	Fevang 2002; Fevang 2004; Ten Broek 2013; Jeppesen 2016
NOM is preferred unless emergency signs are present.	✗	✓	✗	IIC	Fevang 2002; Fevang 2004; Ten Broek 2013; Jeppesen 2016
NOM can safely continue for 72 h.	✗	✓	✓	IIB	Keenan 2014; Sakakibara 2007
Nutritional status and laboratory tests (blood count, lactate, etc.) should be part of the initial evaluation.	✓	✓	✓	IID	Expert opinion
Plain X-rays are not recommended for diagnosis.	✓	✓	✓	IIC	Maglinte 1996
Optimal diagnostic work-up includes CT and water-soluble oral contrast.	✓	✓	✓	IB	Ceresoli 2016; Branco 2010; Abbas 2005; Goussous 2013
Follow-up abdominal X-rays 24 h after contrast to assess resolution.	✗	✓	✓	IB	Ceresoli 2016; Branco 2010; Abbas 2005; Goussous 2013
Water-soluble contrast predicts the need for surgery and reduces hospital LOS.	✗	✓	✓	IIB	Keenan 2014; Sakakibara 2007

WSES, World Society of Emergency Surgery; SBO, small bowel obstruction DTS, direct to surgery; NOM, non-operative management NOM-T, surgery after an unsuccessful appropriate trial of non-operative management; LOS, length of stay.

Resource limitations, regional healthcare delivery differences, and competing clinical priorities may hinder guideline adoption^8^. While deviations from guideline-based care may contribute to population-level outcome disparities, the causes and effects of non-compliance for an individual patient remain poorly characterized^9^. Furthermore, evaluating concordance with guideline-informed care helps shape future guideline development to detail new knowledge, highlight evidence-to-practice gaps, identify implementation barriers, and refine deployment strategies that improve adoption and utilization^10,11^.

Prospective, multicentre, time-bound observational studies, termed snapshot audits, are well suited to assess guideline adherence in heterogeneous real-world environments, identifying specific evidence-to-practice gaps and barriers to implementation^12^. This study, SnapSBO, examines whether and how full or partial guideline compliance influences patient outcomes, including complications and hospital length of stay (LOS).

## Methods

SnapSBO, a prospective, multicentre, time-bound, observational cross-sectional cohort study, was performed using established snapshot audit methodology and was pre-registered with ClinicalTrials.gov (NCT05843097). Centre participation was not limited to specific practice settings or geographical locations^12^. Data were collected over 7 months (1 November 2023 to 31 May 2024), capturing consecutive inpatient admissions for confirmed adhesive SBO. All centres obtained local institutional review board approval and data complied with European Union General Data Protection Regulation (GDPR; (EU) 2016/679) and Health Insurance Portability and Accountability Act (HIPAA; 1996) standards, as applicable.

**Fig. 1:**
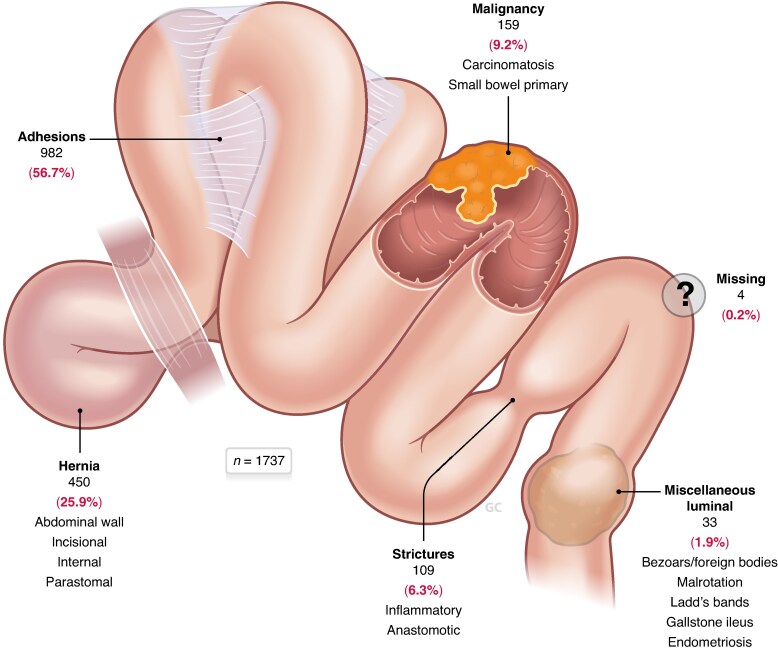
Aetiologies of SBO in the study cohort (*n* = 1737) SBO, small bowel obstruction.

Eligible participants included adults (≥18 years) with radiologically confirmed SBO, defined as an obstruction impeding luminal flow due to a mechanical cause. Patients with functional disease (for example paralytic ileus) and those with incomplete data were excluded from the analysis. As the WSES Bologna guidelines only address adhesive SBO, subgroup analysis of practice patterns was limited to this aetiology. See *Fig. 1*.

Patients were followed from admission to 60 days after discharge to capture complications, recovery milestones, and adherence to guideline recommendations. Primary outcomes included adherence to the Bologna guidelines, complications, LOS, and the incidence of the composite metric ‘optimal outcomes’ (LOS ≤5 days, discharge without complications, and no readmission within 30 days). Secondary outcomes included utilization of guideline elements relevant to the pursued treatment pathway (described below). Variables studied included demographics, co-morbidities, initial treatment strategy (non-operative *versus* operative), diagnostic approach (for example CT and water-soluble enteral contrast use), and institutional characteristics (teaching status and resource level).

Anonymized data were collected using standardized forms and stored in a secure password-protected firewalled Research Electronic Data Capture (REDCap) database (version 14.8.3 - © 2024 Vanderbilt University) compliant with GDPR data-protection standards and housed at the University of Pennsylvania. Local investigators reviewed patient records daily to ensure data fidelity and all sites validated >95% completeness of key fields.

A point-based scoring system provided a consistent framework for assessing compliance with the Bologna guidelines across management pathways. See *Table 2*. Full compliance required utilization of all process-based recommendations; compliance was otherwise assessed as partial compliance or non-compliance. For patients managed using the NOM pathway, compliance was scored on a scale of zero to six points. Elements included appropriate nasogastric tube management, use of water-soluble oral contrast, follow-up abdominal radiography to confirm obstruction resolution, and resolution without surgery (for successful NOM). For patients for whom initial NOM failed (persistent or worsening symptoms after 24 h), compliance required adherence to the same diagnostic and monitoring elements, followed by surgical intervention (thus, such patients underwent surgery after an unsuccessful appropriate trial of NOM (NOM-T)). For patients initially managed using the direct to surgery (DTS) pathway, compliance was scored on a scale of zero to four points based on: completion of laboratory analysis, CT imaging, recognition of the need for emergency surgery, and timely (<6 h) operation.

**Table 2:** WSES Bologna guideline compliance elements captured for the three principal treatment pathways (DTS, NOM-T, and successful NOM)

Treatment pathway	Compliance elements	Scoring (points)
DTS	Nutritional and laboratory tests completed. CT performed. Surgery indicated by clinical/CT signs. Surgery performed without delay.	0–4
NOM-T	Nutritional and laboratory tests completed. CT performed. Appropriate NG tube management. Correct use of water-soluble enteral contrast. Follow-up abdominal X-ray after contrast. Timely surgery after failed NOM.	0–6
Successful NOM	Nutritional and laboratory tests completed. CT performed. Appropriate NG tube management. Correct use of water-soluble oral contrast. Follow-up abdominal X-ray after contrast. SBO resolved without surgery.	0–6

WSES, World Society of Emergency Surgery; DTS, direct to surgery; NOM-T, surgery after an unsuccessful appropriate trial of non-operative management; NOM, non-operative management; NG, nasogastric; SBO, small bowel obstruction.

Descriptive statistics summarize baseline characteristics and adherence rates. Statistical significance was defined as *P* < 0.050. Analyses were performed using Jamovi (version 2.6.2.0, retrieved from https://www.jamovi.org) and the R statistical programming language (version 4.1.2). Figures were created using Biorender^®^ (https://app.biorender.com).

## Results

A total of 70 centres incorporating teaching (94.9%) and community (5.1%) settings in 20 countries across a spectrum of resource availability and socio-economic and cultural contexts were included. Among the 1737 SnapSBO patients, adhesions were the most common aetiology (982 patients (56.7%)).

**Fig. 2:**
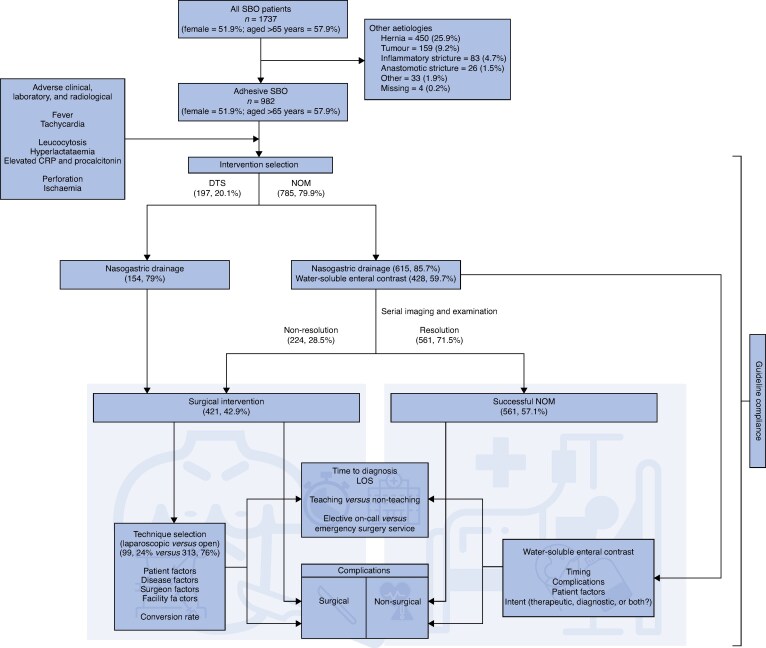
Patient flow diagram SBO, small bowel obstruction; CRP, C-reactive protein; DTS, direct to surgery; NOM, non-operative management.

Patient cohorts were defined by the initial management pathway. See *Fig. 2*. NOM was attempted in 785 of 982 patients (79.9%), with success in 561 patients (71.4% of NOM patients; 57.1% of population). A total of 224 patients (28.5% of NOM patients; 22.8% of overall population) underwent NOM-T and 197 of 982 patients (20.1%) proceeded DTS. Ultimately, 421 patients (42.8%) underwent surgical treatment of adhesive SBO. A nasogastric tube was placed for decompression in 769 patients, including 154 of 197 DTS patients (79%) and 615 of 785 NOM patients (85.7%).

The mean(s.d.) LOS was 4.7(8.5) days for successful NOM (561 patients), 13.0(11.1) days for NOM-T (224 patients), and 8.3(7.2) days for DTS (197 patients) (*P* < 0.001). Optimal outcomes were achieved in 342 of 561 NOM patients (60.1%), 36 of 224 NOM-T patients (16.1%), and 74 of 197 DTS patients (37.6%) (*P* < 0.001). Non-surgical complications, such as aspiration pneumonia, pulmonary embolism, respiratory failure, and acute kidney injury, occurred equally across all cohorts. See *Tables S1–S5*. Decompression via nasogastric tube was not associated with aspiration pneumonia (*P* = 0.092), with 2 events (0.9%) occurring in patients who had a nasogastric tube compared with 25 events (2.5%) occurring in patients who did not have a nasogastric tube.

A total of 25 postoperative complications occurred in NOM-T patients *versus* 23 in DTS patients. The incidence of specific postoperative complications, such as organ space infections, superficial wound dehiscence, and recurrent bowel obstruction, did not differ between the cohorts who were operated on. See *Tables S1*, *S2*. Mortality (Clavien–Dindo grade V) was higher in NOM-T patients (15 of 244 (6.8%)) compared with DTS patients (9 of 197 (4.6%)) (*P* = 0.462).

Full compliance was highest in the NOM group (113 of 656 (17.2%)) when compared with the NOM-T group (24 of 238 (10.1%)) and the DTS group (2 of 477 (0.4%)) (*P* < 0.001). Patients managed with fully guideline-compliant care demonstrated a reduced LOS (mean(s.d.) of 4.1(3.9) days) when compared with patients managed with partial compliant care (mean(s.d.) of 7.8(10) days) or non-compliant care (mean(s.d.) of 11.5(12.2) days) (*P* = 0.002). Similarly, full compliance was associated with more frequent optimal outcomes (full compliance 77.4%, partial compliance 60%, and non-compliance 51.1%) (*P* < 0.001). In DTS patients, progressively higher compliance (scores of 3–4) correlated with fewer complications, with no anastomotic leaks or infections. Lower compliance (scores of 0–2) was linked to increased surgical-site infection (*P* = 0.048) and fascial dehiscence (*P* = 0.019). ICU admission rates and higher revised cardiac risk index (RCRI) scores were inversely associated with compliance across all pathways (*P* < 0.001). See *Tables S1–S5*.

## Discussion

Adhesive SBO has a high recurrence rate and remains a substantial cause of morbidity and mortality that increases healthcare cost^12,13^. Clinical guidelines are generally derived using two principal approaches: using evidence synthesized from prospective RCTs or using expert consensus groups that integrate physiology, existing data, and experience to craft recommendations^14,15^. The WSES Bologna guidelines for adhesive SBO exemplify the latter, providing a pragmatic diagnostic and management framework^3^. Bologna guideline adherence offers the potential to improve morbidity and mortality metrics, reduce healthcare resource utilization, and decrease recurrence of obstruction by promoting consistent and timely interventions. However, such opportunities rely on guideline adoption and utilization.

Other guidelines address patient-specific factors, rather than management approaches. Elderly patients with multi-morbidity, with or without frailty, benefit from individualized risk stratification and goals-of-care determinations that may direct early intervention, NOM, or comfort care^16,17^. Of note, geriatric patient outcomes are not often comprehensively assessed, leaving occurrence rates uncertain^18^. Despite care advances, gaps remain in ensuring goal-concordant care for elderly patients across the panoply of interventions for adhesive SBO. Evidence-informed—as opposed to evidence-based—recommendations, in concert with knowledge and process gaps, underscore the complexity of managing patients with adhesive SBO; surgeons must balance recommendations against individualized and patient-centred care^3,15,18,19^.

RCTs, which actively constrain patient and centre heterogeneity through inclusion criteria, are unable to provide an environmental scan of clinical practice to inform guideline authors or implementation scientists how guideline recommendations are utilized across practice settings^10^. Instead, a prospective, time-bound observational cohort that generates a multicentre international data set is ideally suited to investigate both compliance and outcomes; such studies are termed ‘snapshot audits’^20^. Accordingly, the SnapSBO audit provides a broad opportunity to examine patient-level linkages between contemporary practice patterns, guideline adherence, and their impact on outcomes for patients with adhesive SBO.

These findings reveal a clear association between compliance with the Bologna guidelines and patient outcomes. Greater compliance demonstrated an ordinal association with reduced LOS and more frequent achievement of optimal outcomes across all management pathways. Additionally, greater compliance reduced postoperative complications in DTS and NOM-T patients. These findings underscore the value of reduced variation in care regardless of setting. Institutional factors, such as resource availability and local culture, may strongly influence adherence^21^. At the surgeon level, individual heuristics, clinical experience, and risk tolerance influence the likelihood of guideline-concordant care. The paradoxical increase in complications among fully compliant NOM-T patients likely reflects confounding by indication, wherein more complex cases receive comprehensive and prolonged, but ultimately unsuccessful, NOM before surgery. The absence of significant associations between compliance and non-surgical complications suggests that these events may be primarily driven by co-morbidities, rather than protocolized care adherence. The strong association between ICU admission rates and lower compliance suggests that non-compliance leads to greater clinical deterioration and escalation of care intensity. Additionally, the inverse correlation between compliance and RCRI scores highlights the challenge of achieving full compliance in frail or multi-morbid patients. The observed variability also suggests that, while guidelines offer evidence-informed frameworks, their successful implementation is contingent upon aligning institutional practices and clinician behaviour with care recommendations^22^. Addressing these factors through targeted interventions may reduce practice variability, enhance compliance, and improve patient outcomes^22,23^. Future studies should explore risk-adjusted models to disentangle causality from selection bias, ensuring that compliance-driven improvements in outcomes are accurately interpreted.

Implementation science leverages structured approaches, termed frameworks, to identify barriers to guideline adoption, as well as potential solutions. The updated consolidated framework for implementation research (CFIR 2.0) examines guideline adoption success across domains, such as intervention characteristics (guideline complexity, adaptability, and evidence base), inner settings (key organizational barriers, including local resource limitations and training gaps), outer settings (factors such as policies, funding, and external incentives), and individual behaviours^24^. The expert recommendations for implementing change (ERIC) framework offers a taxonomy of evidence-based strategies to address barriers and facilitate improved guideline adoption^25^. It is essential to recognize that each of these tools is optimally deployed within a single institution or healthcare system, as they benefit from granular input to chart successful paths toward closing the knowledge-to-practice gap.

Barriers at the patient, clinician, and system levels that challenge the implementation of the Bologna guidelines can be examined using the thematic frameworks mentioned above (*Table 3*). Patient-level issues include delayed presentation, often due to limited symptom awareness and access to care^26–28^. This delay is compounded by a lack of adaptability in treatment pathways to accommodate varying degrees of physiological reserve, particularly in frail patients. Addressing these gaps may leverage strategies that include targeted patient awareness campaigns (analogous to those seen promoting breast or colorectal cancer screening), frailty screening tools, and clinician training^28^. Additionally, clinician-level variability in applying laboratory markers, such as lactate or creatinine, and an over-reliance on CT findings for operative decisions highlight the need for multimodal diagnostic frameworks that shape decision-making and sequential management steps^29^. This could be achieved through the integration of decision-support tools into electronic health records (EHRs) or mandatory training modules emphasizing the guidelines’ key recommendations^30–32^. Resource-tiered recommendations, as well as EHR-based compliance dashboards, are among possible solutions that can address systematic barriers to adoption.

**Table 3:** Thematic analysis of potential gaps in guideline adoption and proposed implementation strategies

Level	Gaps and barriers	ERIC implementation approach
Patient	Delayed presentation due to limited awareness of symptoms or access to care.	Engage: Launch targeted awareness campaigns emphasizing early symptom recognition (for example abdominal pain or distension). Prepare: Partner with community health programmes to enhance outreach.
	Invariance in treatment pathways to accommodate degrees of physiological reserve.	Implement: Integrate frailty screening tools (for example the clinical frailty scale) into workflows to individualize care. Evaluate: Assess frailty-specific outcomes to refine guideline use.
Clinician	Underutilization of laboratory findings in surgical decision-making.	Prepare: Provide interactive workshops on integrating lab data into decision-making. Engage: Use clinical vignettes to demonstrate improved outcomes with lab-guided decisions.
	Over-reliance on CT findings for operative decisions.	Implement: Introduce a multimodal diagnostic protocol combining labs, CT, and clinical signs. Engage: Deliver visual decision aids at point of care to reinforce multimodal approaches.
	Variability in adherence due to differences in training, experience, or familiarity with guidelines.	Prepare: Develop standardized training modules on guideline use. Implement: Embed decision support systems in EHRs.
System	Resource constraints in lower-capacity centres limiting guideline application.	Implement: Adapt guidelines with resource-tiered recommendations. Evaluate: Use implementation tracking to measure resource-driven adherence and outcomes.
	Lack of integrated systems to track guideline adherence and evaluate outcomes.	Prepare: Deploy EHR-based compliance dashboards. Implement: Provide real-time feedback to clinicians on adherence and outcomes.
	Insufficient multidisciplinary collaboration, particularly in cases requiring coordinated care.	Engage: Establish cross-discipline case reviews to reinforce collaboration. Implement: Incorporate multidisciplinary rounds into the care workflow with defined team roles.

ERIC, expert recommendations for implementing change; EHR, electronic health record.

System-level challenges, such as resource limitations and inconsistent multidisciplinary collaboration (when of benefit), further hinder guideline adherence. Resource limitations may necessitate deviation from or adaptation of clinical guidelines to embrace feasible alternatives, such as less resource-intensive diagnostics or therapies. It may be ideal to couple adaptations with telemedicine support from tertiary centres, to bridge resource gaps (including specialists) and create opportunities for collaboration around patient care^33^. Additionally, promoting structured care pathways that integrate surgeons, radiologists, and intensivists into interdisciplinary teams can enhance decision-making consistency and quality, as well as improve adherence to evidence-informed practices^34^. Collectively, addressing these barriers requires a tailored implementation strategy. Efforts to educate clinicians, inform and enable patients, and integrate standardized approaches into healthcare systems represent major areas for targeted interventions for healthcare systems that are not limited to the management of SBO. These approaches can be extrapolated to nearly every clinical condition requiring acute inpatient care.

Understanding which elements of a guideline are well embraced—and which ones are poorly adopted—helps direct the content of iterative revisions and deployment approaches. Shifts in medical education to a ‘free, open-access’ approach, implementation tools linked to smart devices, ‘just-in-time’ digital platform training, podcasts, gamification, simulation, and manuscript infographics have largely supplanted traditional didactic-based education^35–37^. Guideline implementation should also incorporate such approaches, especially when compliance is voluntary, as opposed to being directed by a legal mandate that is tied to finances. Specific implementation tools may be ideally suited to drive voluntary compliance.

This study has several limitations that must be acknowledged to contextualize its findings and guide interpretation. As an observational cohort study, causal inferences regarding the impact of guideline adherence on outcomes are limited by the potential for unmeasured confounding. While adjustments were made for known predictors of adverse outcomes, such as laboratory and imaging findings, residual confounding from unmeasured factors, including frailty, time to intervention, or clinician decision-making heuristics, may persist. Variability in data collection across participating centres poses a potential limitation. Despite the use of a standardized protocol and rigorous data validation, differences in local resources, clinician training, and documentation practices could contribute to reporting bias. Patients with high rates of missing data were excluded, which may have introduced bias, by favouring institutions with a more robust data collection infrastructure. The study design assessed guideline compliance using both full compliance and partial compliance as measures, with the latter represented by an unweighted ordinal scale. While this approach captures degrees of adherence, it does not account for the potential heterogeneity in outcomes based on specific combinations of utilized elements in patients who received partially compliant care. For example, some elements may exert a greater influence on outcomes than others, a factor not tested in this analysis. Additionally, the absence of a weighted scoring system means that the relative contributions of individual elements to patient outcomes remain unexplored. This limitation highlights the need for future studies to investigate the impact of specific compliance patterns on clinical outcomes. The generalizability of findings to non-participating regions or healthcare systems is uncertain and requires further study. This study included a diverse range of institutions, but the distribution of academic *versus* non-academic centres, as well as resource variability, may not fully represent global practice patterns. While this analysis identified associations between guideline adherence, surgical intervention, and outcomes, the findings are subject to confounding by indication. Patients requiring surgery often present with more advanced disease, which may independently drive worse outcomes, complicating the interpretation of surgery as a predictor of adverse events.

Guidelines have often been created through consensus statements, reflecting evidence synthesis by a select group of invested content experts^38,39^. Medical professional societies, such as the European Society for Trauma and Emergency Surgery (ESTES) and the WSES, can enhance guideline creation by ensuring that expert consensus panels are diverse regarding their composition. Inclusion of implementation scientists, healthcare administrators, nurses, and patient representatives alongside surgeons helps ensure that guidelines are pragmatic, patient-centred, and contextually relevant^15,40^. Furthermore, representation from low-, middle-, and high-resource settings is crucial for identifying context-specific implementation barriers. Interdisciplinary collaboration with emergency medicine, radiology, anaesthesia, and critical care specialists can address the entire inpatient care continuum. During evidence synthesis, integrating real-world data, such as registry findings and observational studies, alongside RCTs enhances the applicability of recommendations^15^. Future research should adopt a type 2 hybrid implementation-effectiveness design to prospectively evaluate targeted interventions aimed at enhancing Bologna guideline adherence (including, but not limited to, decision-support tools, audit-feedback mechanisms, and clinician education), while concurrently assessing their impact on patient outcomes and sustainability across diverse healthcare settings^41,42^. Clearly graded recommendations, transparent communication of evidence strength, and feasibility assessments during development help ensure that guidelines are evidence-informed and implementable^43,44^.

The findings of SnapSBO’s granular inquiry into the treatment of adhesive SBO at a patient level and across healthcare systems highlight significant discrepancies between evidence-informed expert recommendations and clinical practice. Guideline adherence was associated with shorter LOS and more frequent achievement of optimal outcomes. Nonetheless, overall compliance remained low, particularly in resource-constrained settings. Variations in healthcare infrastructure, clinician decision-making, and patient characteristics likely contribute to the identified evidence-to-practice gaps in the management of adhesive SBO. Addressing these challenges requires a multifaceted approach to ensure guidelines are implementable across diverse healthcare environments.

## Collaborators

Albania: University Hospital of Trauma, TIrana. Agron Dogjani, Kastriot Subashi, Klevis Doci, Joana Spaho. Bahrain: Salmaniya Medical Complex. Ali Abdulla, Sara Ahmed, Yusuf AlAnsari, Mariam AlKooheji, Alaa Marzooq, Khaled Nazzal. Bosnia and Herzegovina: University clinical centre. Emir Ahmetašević, Zlatan Mehmedović, Maja Kovačević, Jasminka Mujkanović. Denmark: Nordsjællands Hospital - University of Copenhagen. Peter Svenningsen, Marie Peter Møller, Gitte Emilje Olsen. Egypt: Cairo University. Abeer Aboalazayem, Muhammad Ashrad Awad, Mahmoud MA Elfiky, Moemen Farouk, Mostafa Gad, Basma Magdy. Estonia: North Estonia Medical Centre. Peep Talving, Edgar Lipping, Edgar Lipping, Sten Saar, Artjom Bahhir, Maarja Talviste. France: Centre Hospitalier Universitaire de Bordeaux. Vincent Dubuisson, Luca Cigagna, Luigi Cayre. Greece: Attikon University Hospital. Spyridon Christodoulou, Panagiotis Kokoropoulos, Ioannis Margaris, Maria Papadoliopoulou, Theodoros A Sidiropoulos, Panteleimon Vassiliu. General Hospital of Nikaia. Evangelos Barkolias, Pavlos Georgalis, Theodosios Kantas, Vasiliki Nikolaou, Aristeidis Papadopoulos, Katerina Tata. Ippokrateio General Hospital. Stergios Arapoglou, Ioannis Gkoutziotis, Aikaterini Mpratko, Elissavet Symeonidou. Laikon General Hospital. Stylianos Kykalos, Nikolaos Machairas, Adam Mylonakis, Panagiotis Sakarellos, Dimitrios Schizas, Michail Vailas. Nafplio General Hospital. Iraklis Anastasiadis, Parmenion Patias, Koumarelas Konstantinos, Mourtarakos Saradis. Ireland: Beaumont Hospital. Charles Lee, Chloe Spillane, Dylan Viani Walsh, Nadia Walsh, Thomas Noel Walsh. Connolly Hospital Blanchardstown. Gabriel Orsi, Andrew Keane, David Kearney, Emma de Sousa. Letterkenny University Hospital. Michael Sugrue, Anne Marie Doyle, Robert Fitzsimmons, Angus J Lloyd, Mohammad Saad Qasim, Mashood Ahmed. St James Hospital. Taylor Jacoby, Michael E Kelly, Shafagh Khodadi, Paul McCormick, Éanna J Ryan, Mahmoud M Salama. St Vincents University Hospital. Helen Heneghan, Cian Davis, Odhran K Ryan, Sean T Martin. Israel: Hadassah Medical Center and Faculty of Medicine, Hebrew University of Jerusalem. Miklosh Bala. Italy: ASST Grande Ospedale Metropolitano Niguarda. Michele Altomare, Stefano PB Cioffi, Andrea Spota, Giada Panagini, Laura Benuzzi, Stefania Cimbanassi. Azienda Ospedaliera San Camillo Forlaninin. Noemi DiFuccia, Stefano Manfroni. Cattinara University Hospital. Alan Biloslavo, Paola Germani, Nicolo de Manzini, Manuela Mastronardi, Anna Modica, Serena Scomersi. Fondazione IRCCS Ca’ Granda Ospedale Maggiore Policlinico. Gabriele Bellio, Luigi Cayre. Fondazione Policlinico Universitario A Gemelli IRCCS. Gaia Altieri, Pietro Fransvea, Gabriele Sganga, Silvia Tedesco. IRCCS Humanitas Research Hospital. Francesca Bunino, Sabrina Caspani, Daniele DelFabbro, Simone Giudici, Giulia Mauri, Paolo Meneghesso. IRCCS Ospedale San Raffaele. Enrico Ortolano, Antonella D'addiego, Francesca Di Vittorio, Gabriele Bormolini, Michele Carlucci. Ospedale Alesandro Manzoni. Giovanni Pesenti, Claudia Tintori, Mauro Zago. Policlinico Umberto I. Martina Zambon, Simona Meneghini, Andrea Mingoli, Giulia Duranti, Gioia Brachini, Pierfrancesco Lapolla. University Hospital. Mehdi Hanafi. México: Nuevo Hospital Civil de Guadalajara "Dr. Juan I. Menchaca". Clara Valdez Cruz, Andrea Alfredo Huerta de León, Jose García Regalado, Pasquale de Jesús Cristiano Nakhal, Diego Enrique Rodríguez González. Hospital Regional de Alta Especialidad del Bajío. Jose Ruiz, Salvador Lozada Jimenez, Oscar Carlos Delgado, Monserrat Reyes Zamorano, Anyely Fuertes Muñoz. Nigeria: Afe Babalola University Multisystem Hospital. Ademola Adetoyese Adeyeye, Ehis Afeikhena, Akinola Akinmade, Babatunde Mustapha. Österreich: Paracelsus Medical University. Jaroslav Presl, Patrick Rebnegger, Bjoern Rudisch, Gruenfelder Johanna, Rokitte Karin. Portugal: Centro Hospitalar Tondela. Filipa M CorteReal, Jorge A Pereira, Joao L Pinheiro, Daniela M Pinto, Andreia J Santos, Andreia M Silva. Hospital Garcia de Orta. Susana Henriques, Joao Melo, António Miguel Pereira, Antonio Miguel Pereira. Hospital da Horta, EPER. Ana Margarida Cabral, Bruno Dias Couto, Barbara Nunes Gama, Catarina Santos Rodrigues. Unidade Local de Saúde de Matosinhos - Hospital Pedro Hispano. Mara Nunes, Bruno Ribeiro Silva, Daniela Tavares, Daniela Tavares. Romania: ‘Constantin Papilian’ Emergency Clinical Military Hospital of Cluj-Napoca. Toma Mihai, Oprea C Valentin. Serbia: General Hospital Vršac. Srdjan S Putnik. General Hospital Đorđe Joanović. Petar Andjic, Marija Djujic, Rastislav Filko, Vanja Kunkin, Andjela Milak, Aleksandar Ognjenovic. Zemun. Nebojsa Mitrovic, Goran Aleksandric. Medical Academy. Mihailo Bezmarević, Sasa Dragović, Milan Jovanović, Bosko Milev, Miroslav Mitrović, Srdjan Petković. Novi Sad. Valentina Isakovic, Nikola Zoran Nikolic, Predrag Radic, Dragan Luka Vasic. Clinical Center of Serbia. Zlatibor M Loncar, Dusan D Micic, Vladimir R Resanovic, Pavle D Vladimir. Clinical Center of Serbia; Medical Faculty, University of Belgrade. Krstina S Doklestic Vasiljev. University Clinical Hospital Center "Zvezdara" Clinic for Surgery "Nikola Spasic". Ljiljana Velibor Milic, Vladica Velibor Cuk, Jovan Todor Juloski, Radisav Slavoljub Radulovic, Dragana Dragan Arbutina. Spain: Complejo Asistencial Universitario de Salamanca. Jacobo Trebol, Manuel Torres-Jurado, Andres J Valera-Montiel, Francisco E Blanco-Antona. Hospital Clínico Universitario. Beatriz de Andrés-Asenjo, Maria Ruiz-Soriano, Tania Gómez-Sanz, Andrea Vázquez-Fernández, Juan Beltran de Heredia. Hospital General Universitario Gregorio Marañón. Cristina Rey-Valcárcel, Monica Ballón-Bordon, Maria Pérez-Díaz, Maria Dolores Sanchez-Rodriguez, Jose David Gonzalez-Esteban. Hospital Nuestra Señora de Sonsoles. Celia Alegre Nevado, Ricardo Montenegro Romero. Hospital San Juan de Dios del Aljarafe. Inés Capitán del Río, Hospital Universitari Parc Taulí. Andrea Campos-Serra, Raquel Gracia-Roman, Heura Llaquet-Bayo, Anna Muñoz-Campaña, Giulia Vitiello. Hospital Universitario Donostia. Lorena Apodaca Murguiondo, Inigo Augusto Ponce, Amaia Garcia Dominguez, Aintzane Lizarazu Perez. Hospital Universitario Infanta Cristina. Elena Sagarra Cebolla, Mónica García Aparicio, Paloma Garaulet González, Benito Miguel Josa Martínez, Miriam Fraile Vasallo. Hospital Universitario J. M Morales Meseguer. Mónica MengualBallester, Isabel Andrés Lucas Zamorano, Jose Martinez Moreno, Manuel Luis Buitrago Ruiz, Clara Piñera Morcillo. Universitario Nuestra Señora de Candelaria. Alberto Díaz García, Hanna Hernández Oaknin, Maria Pellicer Barreda, Jennifer Amparo García Niebla, Antonio Pérez Álvarez. Hospital Universitario Príncipe de Asturias. Diego Cordova, Laura Jiménez, Fernando Mendoza, Cristina Vera, Alberto Vilar Tabanera. Hospital Universitario Virgen Macarena. María de los Ángeles Gil-Olarte Márquez, José Antonio López-Ruiz, Mª Estela Romero-Vargas, Julio Reguera-Rosal, Alberto García-García, Beatriz Marenco de la Cuadra. Hospital Universitario Virgen del Rocio. Eduardo Perea del Pozo, Virginia Duran Muñoz, Felipe Pareja Ciuró. Hospital de Mataró. Ainoa Benavides dos Santos, Ernest Bombuy, Anna G-Monferrer, Sandra López Gordo. Hospital de la Merced. José Guerra, Vanessa Sojo, Begona De Soto, Aaron Roman. Hospital del Mar. Ana María González-Castillo, Elena Manzo, Estela Membrilla-Fernandez, Amalia Pelegrina-Manzano, Simone Cremona. La Paz University Hospital. Alexander Forero-Torres, Santiago Valderrabano, Francisco Reinoso Olmedo, Fuad Lopez Fernandez. Urduliz Hospital. Aitor Landaluce-Olavarria, Jon Barrutia- Leonardo, Alba Garcia-Trancho, Melania Gonzalez-De Miguel, Izaskun Markinez-Gordobil. United Arab Emirates: Sheikh Shakhbout Medical City. Maryam Makki, Dana Altamimi, Sadhika Vinod. United Kingdom: Liverpool University Hospitals NHS Foundation TrustNHS. Olga Rutka, John V Taylor. Addenbrooke's Cambridge University Hospital. M Denton, S Gourgiotis, R Ravi, A J Ribbits. University Hospital Wishaw. Jared Wohlgemut, Shehryar Rangana Khan, Christopher Leiberman, Sabreen P Elbakri, Charlie A Edgar. Wirral University Teaching Hospitals NHS Foundation Trust. Conor Magee, Oluwaseun Oyekan, Mehwish Ansar, Jeremy Wilson, Rahel Rashid. United States: Grand View Health. Deborah Atwell, Joshua Cassedy, Brianna Gabriel, William Hoff, Shyam Murali. University of Pennsylvania. Anna E Garcia Whitlock, Carolyn Susman, Sarah Barnett, Emily Ertmann, Camden DeSanctis, Pavel Karasek, Nathan Klingensmith. University of Texas Southwestern Medical Center. Dale F Butler, Brandon Bruns, Ankeeta Mehta, Vanessa Nomellini, Keyus Patel, Anthony Tannous.

## Supplementary Material

znaf080_Supplementary_Data

## Data Availability

The data sets generated and analysed during this study are available from the corresponding author, on behalf of the study steering group, upon reasonable request.

## Supplementary material

*Supplementary*  *material* is available at *BJS* online.
