# Supplementary material for: Small bowel obstruction outcomes according to compliance with the World Society of Emergency Surgery Bologna guidelines
Source: Br J Surg. 2025 Apr 18;112(4):znaf080. doi: 10.1093/bjs/znaf080 (PMC12005947; doi:10.1093/bjs/znaf080)
Supplement: znaf080_Supplementary_Data [file znaf080_supplementary_data.docx]

**Title:** Small bowel obstruction outcomes according to compliance with WSES Bologna Guidelines

Lewis J. Kaplan^1^, Isidro Martinez-Casas^2^, Shahin Mohseni^3^, Matteo Cimino^4^, Hayato Kurihara^4^, Matthew J. Lee^5^, *and* Gary A. Bass^1^ *for the SnapSBO Collaborators*.*

1. Perelman School of Medicine, University of Pennsylvania; Division of Traumatology, Surgical Critical Care and Emergency Surgery; Philadelphia, PA; USA.
2. Unidad de Cirugía de Urgencias y Trauma del Hospital Universitario Virgen del Rocio, Sevilla, Spain.
3. School of Medical Sciences, Orebro University, Orebro, Sweden
4. Department of Emergency Surgery, Fondazione IRCCS Ca’ Granda Ospedale Maggiore Policlinico, Milan, MI, Italy.
5. Institute for Applied Health Research, University of Birmingham, Birmingham, United Kingdom.

**Corresponding Author:**

Gary Alan Bass, MD MSc MBA PhD FEBS(Em Surg)

Assistant Professor of Surgery,

Perelman School of Medicine, University of Pennsylvania,

Division of Traumatology, Surgical Critical Care and Emergency Surgery Philadelphia, PA; USA.

Email: gary.bass@pennmedicine.upenn.edu

ORCID: 0000-0002-1918-9443

**Supplementary Materials - Index**

| **Supplementary Figures and Tables** |  |
| --- | --- |
| Supplementary Table 1 | *2* |
| Supplementary Table 2 | *2* |
| Supplementary Table 3 | *3* |
| Supplementary Table 4 | *4* |
| Supplementary Table 5 | *5* |

**Supplementary Figures and Tables**

**Supplemental Tables**

WSES Bologna Guideline compliance was scored on a scale of 0 to 4 points for DTS, based on four elements: *completion of laboratory analysis, CT imaging, emergency surgical indication recognition (e.g., peritonitis, pneumatosis, bowel ischemia), and timely (< 6 hours) surgical intervention*. In the NOM with intention to treat cohorts (both successful NOM and surgery after trial NOM-T), guideline compliance was scored on an ordinal scale of 0 to 6 points, based on six elements: *no indication for DTS, appropriate nasogastric tube management, use of water-soluble oral contrast to predict surgical needs, follow-up abdominal radiography to confirm obstruction resolution, and resolution without surgery (for successful NOM) or prompt surgical intervention (<24 hours)*.

*Supplemental Table 1 - Graded compliance (0 = no compliance, 4= full compliance) in DTS patients vs. post-operative surgical complication incidence; n(%)*

|  | **DTS patient-level graded compliance** | | | | | |  |  |
| --- | --- | --- | --- | --- | --- | --- | --- | --- |
| **Surgical Complications** |  | **0** | **1** | **2** | **3** | **4** | **Total** | ***p*** |
| anastomotic leak |  | 1 (2.7) | 1 (0.3) | 3 (0.6) | 0 (0.0) | 0 (0.0) | 5 (0.5) | *0.454* |
| surgical site infection |  | 1 (2.7) | 3 (0.8) | 23 (4.4) | 0 (0.0) | 0 (0.0) | 27 (2.9) | ***0.048*** |
| intraperitoneal abscess |  | 1 (2.7) | 6 (1.7) | 10 (1.9) | 0 (0.0) | 0 (0.0) | 17 (1.9) | *0.992* |
| superficial wound dehiscence |  | 3 (8.1) | 4 (1.1) | 18 (3.5) | 0 (0.0) | 0 (0.0) | 25 (2.7) | *0.071* |
| fascial dehiscence |  | 3 (8.1) | 3 (0.8) | 8 (1.5) | 0 (0.0) | 0 (0.0) | 14 (1.5) | ***0.019*** |
| post operative hemorrhage |  | 0 (0.0) | 5 (1.4) | 7 (1.3) | 1 (50.0) | 0 (0.0) | 13 (1.4) | ***<0.001*** |
| post-op SBO |  | 2 (5.4) | 9 (2.5) | 8 (1.5) | 0 (0.0) | 0 (0.0) | 19 (2.1) | *0.528* |
| ICU admission |  | 11 (30.6) | 43 (12.9) | 52 (10.4) | 2 (100.0) | 0 (0.0) | 108 (12.4) | ***<0.001*** |

Supplemental Table 2 - Graded compliance (0 = no compliance, 6 = full compliance) in NOM-T patients vs. post-operative surgical complication incidence; n(%)

|  |  | | **NOM-T patient-level graded compliance** | | | | | | | |  | | | | | | | |  | |  |
| --- | --- | --- | --- | --- | --- | --- | --- | --- | --- | --- | --- | --- | --- | --- | --- | --- | --- | --- | --- | --- | --- |
| **Surgical Complications** |  | | **0** | | **1** | | **2** | | **3** | | **4** | | **5** | | **6** | | **Total** | | ***p*** | |  |
| anastomotic leak |  | | 1 (14.3) | | 1 (1.4) | | 1 (0.5) | | 0 (0.0) | | 1 (0.5) | | 0 (0.0) | | 0 (0.0) | | 4 (0.5) | | ***<0.001*** | |  |
| surgical site infection |  | | 1 (14.3) | | 1 (1.4) | | 8 (4.1) | | 0 (0.0) | | 5 (2.5) | | 3 (2.3) | | 5 (26.3) | | 23 (2.9) | | ***<0.001*** | |  |
| intraperitoneal abscess |  | | 0 (0.0) | | 1 (1.4) | | 5 (2.6) | | 0 (0.0) | | 5 (2.5) | | 1 (0.8) | | 1 (5.3) | | 13 (1.6) | | *0.289* | |  |
| superficial wound dehiscence |  | | 1 (14.3) | | 1 (1.4) | | 7 (3.6) | | 0 (0.0) | | 5 (2.5) | | 4 (3.1) | | 3 (15.8) | | 21 (2.6) | | ***0.001*** | |  |
| fascial dehiscence |  | | 1 (14.3) | | 1 (1.4) | | 4 (2.1) | | 0 (0.0) | | 2 (1.0) | | 1 (0.8) | | 2 (10.5) | | 11 (1.4) | | ***<0.001*** | |  |
| post operative hemorrhage |  | | 0 (0.0) | | 1 (1.4) | | 7 (3.6) | | 0 (0.0) | | 4 (2.0) | | 1 (0.8) | | 0 (0.0) | | 13 (1.6) | | *0.175* | |  |
| post-op SBO |  | | 0 (0.0) | | 1 (1.4) | | 4 (2.1) | | 1 (0.5) | | 3 (1.5) | | 0 (0.0) | | 0 (0.0) | | 9 (1.1) | | *0.658* | |  |
| ICU admission | | |  | | 3 (42.9) | | 17 (25.4) | | 30 (16.3) | | 16 (8.9) | | 16 (8.1) | | 7 (5.6) | | 4 (22.2) | | 93 (11.9) | | ***<0.001*** |
|  |  | |  | |  | |  | |  | |  | |  | |  | |  | |  | |  |

Supplemental Table 3 - Graded compliance (0 = no compliance, 6 = full compliance) in NOM patients vs. non-surgical complication incidence; n(%)

|  |  | **NOM patient-level graded compliance** | | | |  | | | |  |
| --- | --- | --- | --- | --- | --- | --- | --- | --- | --- | --- |
| **Non-surgical Complications** |  | **0** | **1** | **2** | **3** | **4** | **5** | **6** | **Total** | ***p*** |
| aspiration pneumonia |  | 0 (0.0) | 2 (3.1) | 4 (2.5) | 4 (3.1) | 6 (2.8) | 6 (2.9) | 2 (2.2) | 24 (2.8) | *0.998* |
| pulmonary embolism |  | 0 (0.0) | 2 (33.3) | 1 (5.0) | 0 (0.0) | 1 (4.5) | 1 (5.9) | 0 (0.0) | 5 (6.2) | *0.123* |
| myocardial infarction |  | 0 (0.0) | 0 (0.0) | 1 (5.0) | 0 (0.0) | 0 (0.0) | 2 (11.8) | 0 (0.0) | 3 (3.8) | *0.442* |
| cerebrovascular accident |  | 0 (0.0) | 0 (0.0) | 0 (0.0) | 2 (15.4) | 1 (4.5) | 1 (5.6) | 0 (0.0) | 4 (4.9) | *0.466* |
| renal failure |  | 0 (0.0) | 1 (16.7) | 5 (25.0) | 4 (33.3) | 7 (30.4) | 4 (23.5) | 0 (0.0) | 21 (25.6) | *0.802* |
| respiratory failure |  | 0 (0.0) | 2 (33.3) | 6 (28.6) | 4 (36.4) | 8 (34.8) | 5 (29.4) | 2 (40.0) | 27 (32.5) | *0.993* |
| post operative delirium |  | 0 (0.0) | 1 (16.7) | 3 (13.6) | 3 (25.0) | 5 (21.7) | 5 (27.8) | 1 (25.0) | 18 (21.2) | *0.920* |
| ICU admission |  | 3 (42.9) | 17 (27.9) | 33 (21.3) | 25 (20.5) | 17 (8.1) | 10 (5.0) | 0 (0.0) | 105 (12.5) | **<0.001** |

**Supplemental Table 4 -** Graded compliance in DTS (0 = no compliance, 4 = full compliance) patients vs. non-surgical complication incidence; n(%)

|  | | **DTS patient-level graded compliance** | | | | | | | | | | |  | |  | |  |
| --- | --- | --- | --- | --- | --- | --- | --- | --- | --- | --- | --- | --- | --- | --- | --- | --- | --- |
| **Non-surgical complications** | |  | | **0** | **1** | | **2** | | **3** | | **4** | | **Total** | | ***p*** | |  |
| aspiration pneumonia | |  | | 0 (0.0) | 7 (2.0) | | 15 (2.9) | | 1 (50.0) | | 0 (0.0) | | 23 (2.5) | | ***<0.001*** | |  |
| pulmonary embolism | |  | | 0 (0.0) | 3 (10.3) | | 2 (4.2) | | 0 (0.0) | | 0 (0.0) | | 5 (6.2) | | *0.709* | |  |
| myocardial infarction | |  | | 0 (0.0) | 0 (0.0) | | 2 (4.2) | | 1 (50.0) | | 0 (0.0) | | 3 (3.8) | | ***0.005*** | |  |
| cerebrovascular accident | |  | | 0 (0.0) | 3 (9.7) | | 1 (2.1) | | 0 (0.0) | | 0 (0.0) | | 4 (4.9) | | *0.475* | |  |
| renal failure | |  | | 1 (100.0) | 10 (33.3) | | 11 (22.9) | | 0 (0.0) | | 0 (0.0) | | 22 (26.8) | | *0.303* | |  |
| respiratory failure | |  | | 0 (0.0) | 6 (20.0) | | 20 (40.0) | | 0 (0.0) | | 1 (100.0) | | 27 (32.5) | | *0.091* | |  |
| post operative delirium | |  | | 0 (0.0) | 7 (21.9) | | 12 (24.0) | | 0 (0.0) | | 0 (0.0) | | 19 (22.4) | | *0.814* | |  |
| ICU admission | |  | | 11 (30.6) | | | 43 (12.9) | | 52 (10.4) | | 2 (100.0) | | 0 (0.0) | | 108 (12.4) | | ***<0.001*** |

**Supplemental Table 5 -** Graded compliance (0 = no compliance, 6 = full compliance) in NOM-T patients vs. non-surgical complication incidence; n(%)

|  |  | **NOM-T patient-level graded compliance** | | | | | | |  |  |
| --- | --- | --- | --- | --- | --- | --- | --- | --- | --- | --- |
| **Non-surgical Complications** |  | **0** | **1** | **2** | **3** | **4** | **5** | **6** | **Total** | ***p*** |
| aspiration pneumonia |  | 0 (0.0) | 2 (2.9) | 5 (2.6) | 5 (2.7) | 3 (1.5) | 6 (4.7) | 0 (0.0) | 21 (2.6) | *0.693* |
| pulmonary embolism |  | 0 (0.0) | 2 (33.3) | 1 (5.3) | 1 (8.3) | 1 (6.2) | 0 (0.0) | 0 (0.0) | 5 (7.5) | *0.212* |
| myocardial infarction |  | 0 (0.0) | 0 (0.0) | 1 (5.3) | 0 (0.0) | 1 (6.2) | 0 (0.0) | 0 (0.0) | 2 (3.0) | *0.861* |
| cerebrovascular accident |  | 0 (0.0) | 0 (0.0) | 0 (0.0) | 2 (15.4) | 0 (0.0) | 0 (0.0) | 1 (33.3) | 3 (4.4) | ***0.034*** |
| renal failure |  | 0 (0.0) | 1 (16.7) | 4 (21.1) | 2 (15.4) | 6 (37.5) | 3 (25.0) | 0 (0.0) | 16 (23.5) | *0.690* |
| respiratory failure |  | 0 (0.0) | 2 (33.3) | 7 (35.0) | 2 (15.4) | 4 (25.0) | 5 (38.5) | 1 (50.0) | 21 (30.0) | *0.760* |
| post operative delirium |  | 0 (0.0) | 1 (16.7) | 3 (14.3) | 2 (14.3) | 4 (25.0) | 2 (15.4) | 2 (66.7) | 14 (19.2) | *0.369* |
| ICU admission |  | 3 (42.9) | 17 (25.4) | 30 (16.3) | 16 (8.9) | 16 (8.1) | 7 (5.6) | 4 (22.2) | 93 (11.9) | ***<0.001*** |
